# Effect of Low Temperature on Content of Primary Metabolites in Two Wheat Genotypes Differing in Cold Tolerance

**DOI:** 10.3390/metabo14040199

**Published:** 2024-04-03

**Authors:** Alexander Deryabin, Kseniya Zhukova, Natalia Naraikina, Yuliya Venzhik

**Affiliations:** K. A. Timiryazev Institute of Plant Physiology, Russian Academy of Sciences, Moscow 127276, Russia; zhukova@ifr.moscow (K.Z.); naraikina@ifr.moscow (N.N.); jul.venzhik@ifr.moscow (Y.V.)

**Keywords:** *Triticum aestivum*, adaptation, chilling, *COR*-genes, growth, lipid peroxidation, photosynthetic apparatus, proteins, sugars

## Abstract

The study of cold-tolerance mechanisms of wheat as a leading cereal crop is very relevant to science. Primary metabolites play an important role in the formation of increased cold tolerance. The aim of this research is to define changes in the content of primary metabolites (soluble proteins and sugars), growth, and photosynthetic apparatus of freezing-tolerant and cold-sustainable wheat (*Triticum aestivum* L.) genotypes under optimal conditions and after prolonged (7 days) exposure to low temperature (4 °C). In order to gain a deeper comprehension of the mechanisms behind wheat genotypes’ adaptation to cold, we determined the expression levels of photosynthetic genes (*RbcS*, *RbcL*) and genes encoding cold-regulated proteins (*Wcor726*, *CBF14*). The results indicated different cold-adaptation strategies of freezing-tolerant and cold-sustainable wheat genotypes, with soluble proteins and sugars playing a significant role in this process. In plants of freezing-tolerant genotypes, the strategy of adaptation to low temperature was aimed at increasing the content of soluble proteins and modification of carbohydrate metabolism. The accumulation of sugars was not observed in wheat of cold-sustainable genotypes during chilling, but a high content of soluble proteins was maintained both under optimal conditions and after cold exposure. The adaptation strategies of wheat genotypes differing in cold tolerance were related to the expression of photosynthetic genes and genes encoding cold-regulated proteins. The data improve our knowledge of physiological and biochemical mechanisms of wheat cold adaptation.

## 1. Introduction

The study of plant adaptation mechanisms to abiotic stressors is one of the priorities of modern biological research. Low temperature is the most common of the environmental stresses that seriously affect the growth and development of plants [1]. Low-temperature stress may be further categorized into chilling stress (0–15 °C) and freezing stress (<0 °C). On Earth, approximately 64% of the land is subject to a seasonal drop in temperature below 0 °C [2]; meanwhile, 26% of this territory is used for agricultural plant cultivation [3]. The frequency of sudden cold spells is increasing, and this significantly reduces crop yields [4]. Plants are sustainable in low temperatures throughout their life, from seed germination to maturation. During evolution, plants have evolved morphological, physiological, biochemical, and molecular mechanisms of temperature adaptation. Tolerance to low temperatures varies among plant species, and it is likely that different mechanisms are activated in different genotypes.

Hexaploid wheat (*Triticum* spp.) is one of the leading cereal crops grown in a vast amount of fertile land from the northern polar regions to South America and Africa. Based on its growth patterns, wheat (*Triticum aestivum* L.) is divided into winter wheat (freezing-tolerant) and spring wheat (cold-sustainable) [5]. Wheat is grown for its seed, which is a staple food product for all mankind. Wheat grains are a source of vegetable proteins, fats, fiber, minerals, vitamins, and carbohydrates [6]. Alterations in the environmental conditions have a profound effect on the metabolic activities of wheat [7]. In spring, freezing and sustainable genotypes of wheat seedlings are often exposed to low temperatures (spring frosts), which leads to reduced grain yields [8,9]. A decrease in grain yield can be primarily associated with the effect of cold on growth processes, and, especially, on the photosynthetic apparatus. This could lead to the degradation of ribulose-1,5-bisphosphate carboxylase/oxygenase (RuBisCo) and a decrease in the photosynthetic capacity of the leaves, as well as the transportation of primary metabolites–carbohydrates [10,11]. Mechanisms of wheat-plant tolerance to low temperatures are actively studied. It is important to research strategies for increasing wheat cold tolerance, as well as to create new genotypes and expand planting areas [12]. 

The increase in resistance to low temperatures of wheat freezing-tolerant genotypes under chilling is accompanied by complex adaptive changes. Such wheat plants are able to maintain a stable content of photosynthetic pigments, RuBisCo activity, and photosynthesis intensity under low-temperature conditions and, as a result, accumulate compatible sugars necessary for the process of cold adaptation [13,14]. Under the influence of a cold temperature, the cell membranes of adapted plants become more fluid, due to an increase in the content of unsaturated fatty acid. What is more, it promotes the accumulation of compatible osmolytes and activation of components of the antioxidant system— enzymes (superoxide dismutase, catalase, peroxidases, etc.), low molecular weight antioxidants (ascorbate, glutathione, carotenoids, sugars, etc.) and alteration of the expression of genes encoding the synthesis of structural and enzymatic proteins [15,16,17,18]. Low temperature modifies the composition of primary metabolites (proteins, carbohydrates, lipids, organic acids, etc.) that are essential for plant growth and development [19,20,21]. Unlike secondary metabolites (phenolic compounds, terpenoids, alkaloids, etc.), primary metabolites are not species-specific, but play a role in the interaction of the plant with its environment [22,23]. In cold-sustainable plants, low temperature induces a general metabolic switch involving many negative changes that can induce oxidative stress by increasing reactive oxygen species (ROS) generation, lipid peroxidation (LPO) intensity and cytoskeletal rearrangement [24].

Wheat genotypes differ in a number of genetic features. It is thought that in cold-sustainable (spring) genotypes, dominant vernalization genes (*Vrn1*, *Fr1*) limit the expression of genes encoding enzymes of the antioxidant system [25] and *COR*-genes (Cold-Regulated Genes) [26]. In freezing-tolerant (winter) wheat genotypes, low temperature “turns on” a powerful response to cold stress, including the expression of a number of *COR*-genes and related proteins [26,27].

Soluble sugars are among the most important primary metabolites providing polyfunctional protective effects on plants under low temperature conditions [28,29]. Compatible carbohydrates control plant ontogenesis through activation/repression of genes regulating such physiological and biochemical processes as growth, photosynthesis, and biosynthesis of other primary metabolites [30,31]. For example, in late fall, the number of soluble sugars in tillering nodes of some cereals can exceed 50% of the dry weight [32]. *Arabidopsis thaliana* (ecotype Columbia), a model plant species for biologists, accumulation of sucrose, glucose, and fructose begins as early as 1–4 h after chilling. The content of these sugars was positively correlated with *Arabidopsis’s* cold tolerance [33,34]. Field experiments conducted during winter in China showed a predominance of sugars in the tissues of winter genotypes, in contrast to spring genotypes [25]. The authors opine that the high cold tolerance of winter wheat varieties may be associated with effective osmoregulation ability and high photosynthetic capacity. However, there is evidence of a low positive correlation between the accumulation of sugars and the level of cold tolerance in two ryegrass genotypes [35]. In our study different cold adaptation strategies of freezing-tolerant and cold-sustainable wheat genotypes were indicated, with soluble proteins and sugars playing a significant role in this process.

The aim of this study is to define changes in the growth, photosynthetic apparatus and content of primary metabolites (soluble proteins and sugars) in leaves of freezing-tolerant genotype Moskowskaya 39 and cold-sustainable genotype Zlata of wheat under optimal conditions and after chilling (4 °C, 7 d). In order to gain a deeper comprehension of the mechanisms behind wheat genotypes’ adaptation to cold, we determined the expression levels of photosynthetic genes (*RbcS*, *RbcL*) and genes encoding cold-regulated (COR) proteins (*Wcor726*, *CBF14*).

## 2. Materials and Methods

### 2.1. Plant Material and Growth Conditions

Wheat plant seeds (*Triticum aestivum* L., Poaceae) were used in this study. The seeds of freezing-tolerant genotype Moskovskaya 39 (M39) and cold-sustainable genotype Zlata (Zl) were supplied by the Federal Research Center “Nemchinovka” (Novoivanovskoye, Russia). The seeds were germinated in distilled water at a temperature of 22 °C and a photoperiod of 16 h, illumination 100 μmol photons · m^−2^ s^−1^. After reaching 10 days, seedlings were placed in a KBW-240 climatic chamber (Binder, Tuttlingen, Germany) at 4 °C for 7 days, keeping other conditions unchanged.

### 2.2. The Length of the First Leaf and Dry Weight

The length of the first leaf was measured at 10 days of growth, as well as after cold treatment (4 °C, 7 days) at 17 days.

Leaf dry weight of 10-day and 17-day-old seedlings was determined after drying in a thermostat at 100 ± 5 °C to constant weight and was expressed as a percentage of the initial wet weight of the sample.

### 2.3. Malondialdehyde (MDA) Content

LPO level in the leaves was determined as the content of MDA as described by Heath and Packer [36] with minor modifications. Leaf sample (≈300 mg) was homogenized in 5 mL of 0.35 M NaCl (Reachem, Moscow, Russia) in 0.1 M Tris-HCl buffer (Reachem, Moscow, Russia), pH 7.6. The homogenate (3 mL) was mixed with 2 mL of 0.5% thiobarbituric acid (Merck SA, Darmstadt, Germany) in 20% trichloroacetic acid (Reachem, Moscow, Russia) and then heated at 100 °C for 30 min, cooled, and filtered. The extraction medium containing the reagent was used as a control. A Genesys 10 UV spectrophotometer (Termo Electron Corporation, Waltham, MA, USA) was used in the study. Measurements were corrected for nonspecific absorbance by subtracting the values obtained at 532 nm and 600 nm. The concentration of MDA was calculated from molar extinction: $C=\frac{D}{\varepsilon l}$, where *C* is the concentration of MDA, μM; *D* is optical density; *ε* is the molar extinction coefficient (*ε* = 1.56·10^5^ M^−1^ cm^−1^); and *l* is cuvette (1.0 cm). The content of MDA was calculated in μM/g dry weight of leaves.

### 2.4. Photosynthetic Pigments Content

The content of chlorophyll *a* (Chl *a*), chlorophyll *b* (Chl *b*), and total carotenoids (*Car*) in the leaves was determined spectrophotometrically at wavelengths corresponding to the absorption maxima of Chl *a*, Chl *b* and *Car* in an 80% acetone (Reachem, Moscow, Russia) solution—663 nm, 646 nm and 470 nm, respectively. Pigment concentrations were calculated using the following formulas [37]:

C*_a_* = 12.21*D*_663_ − 2.81*D*_646_, C*_b_* = 20.13*D*_646_ − 5.03*D*_663_, C*_car_* = (1000*D*_470_ − 3.27C*_a_* − 104C*_b_*)/198.

The pigment content was expressed in mg/g dry weight of leaves.

We calculated the chlorophyll portion in a light-harvesting complex (LHC), assuming that practically all Chl *b* was located in LHC and the chlorophyll ratio in this complex was equal to 1.2 [38]. The pigment content was expressed as mg/g dry weight of leaves.

### 2.5. Soluble Sugars (Glucose, Fructose, and Sucrose) Content

Weighed samples (500 mg) of leaves were fixed with 96% boiling ethanol (Reachem, Moscow, Russia). Tissue was homogenized and sugars were extracted with 80% ethanol three times. In the obtained extracts the content of fructose was determined according to Roe followed by conversion to sucrose content [39]. The glucose content was determined by the glucose oxidase method using the Olvex diagnosticum Kit (Vital Diagnostics, St Petersburg, Russia).

### 2.6. The Total Soluble Protein Content

Leaf samples (500 mg) were homogenized in isolation medium (50 mM Tris-HCl, pH 7.6, 3 mM EDTA, 250 mM sucrose, 3.6 mM cysteine, 5 mM ascorbic acid, 3 mM MgCl_2_, 2 mM DTT, 2 mM PMSF) (Reachem, Moscow, Russia), and centrifuged for 20 min at 16,000× *g*. The extract was purified on a PD-10 midiTrap G-25 column (GE Healthcare, Chicago, IL, USA). The protein content in the supernatant was determined spectrophotometrically at 562 nm using the Bicinchoninic Acid Kit for Protein Determination (BCA1-1KT, Sigma-Aldrich, Saint Louis, MO, USA), according to the manufacturer’s protocol.

### 2.7. Freezing Tolerance of Plants

The survival rate of wheat seedlings was assessed after freezing in a climatic chamber MIR-153 (Sanyo, Tokyo, Japan) at temperatures of 0 °C, −3 °C, −5 °C, and −7 °C for 24 h. Then the plants were kept for 1 day at 4 °C in the dark, after which they were transferred to normal conditions (22 °C, daylight) for 72 h. The survival rate was judged by the number of undamaged seedlings as a percentage of the total number of frozen plants.

### 2.8. Total RNA Extraction, cDNA Synthesis and Gene Expression by Real-Time Quantitative PCR

Total RNA was isolated from 50 mg of leaf tissue samples by using the Spectrum Plant Total RNA Kit (Sigma-Aldrich, USA) according to the manufacturer’s instructions with modification of the homogenization step [40]. The quality and quantity of purified RNA were determined by a NanoDrop-2000 spectrophotometer (Thermo Fisher Scientific, Wilmington, DE, USA)and analyzed by 2% agarose gel electrophoresis. To remove residual genomic DNA impurities, total RNA preparations were treated with DNase I (Thermo Fisher Scientific, Waltham, MA, USA). cDNA was synthesized using a reverse transcription kit RevertAid (Thermo Fisher Scientific, USA) with included primers *oligo* (*dT*) *21* for nuclear coding genes and *random 6* for chloroplast coding genes [41,42].

Real-time quantitative PCR (RT-qPCR) was carried out on a CFX96 Touch™ (Bio-Rad, Hercules, CA, USA), using the SYBR Green I intercalating dye (Evrogen, Moscow, Russia). The reaction mixture for quantitative PCR in a volume of 25 μL contained 5 μL of qPCRmix HS SYBR (Evrogen, Moscow, Russia), 0.2 μM of each primer, and 15 ng of the cDNA template. The following amplification conditions were used: 95 °C for 5 min, followed by 40 cycles of 95 °C for 15 s, 60 °C for 30 s, and 72 °C for 30 s.

### 2.9. Expression Level of COR-Genes and Genes Encoding RuBisCo

Gene-specific primers (Table 1) for amplification of target *COR*-genes (*Wcor726*, *CBF14*) and reference gene *TaRP15* were selected by using Primer-BLAST (https://www.ncbi.nlm.nih.gov/tools/primer-blast/, accessed on 1 February 2024) and OligoAnalyzer™ tool Version 3.1, Integrated DNA Technologies (Coralville, IA, USA) (accessible at https://www.idtdna.com/pages/tools/oligoanalyzer on 3 May 2023). Gene-specific primers for amplification of large (*RbcL*) and small (*RbcS*) subunits of RuBisCo target genes were borrowed from publications [12,43]. Primers were designed to bind to all three wheat sub-genomes [43]. The transcript levels were normalized to the expression of the reference gene *TaRP15* (RNA polymerase I, II, and III, 15kDa subunit) [44]. The relative expression levels were calculated using the Pfaffl method [45]. The level of gene expression in cold-sustainable genotype Zl at a temperature of 22 °C was set to 1 (100%). Each qRT-PCR reaction was performed in three biological and two technical replicates.

### 2.10. Statistical Analysis

One-way analysis of variance (ANOVA) was performed by Origin 7.0 software. Results presented as means of three replicates marked with the same letter were not significantly different at *p* ≤ 0.05 (Tukey’s test). The tables and figures show the mean values of the typical experiment and their standard errors.

## 3. Results

### 3.1. Effect of Low Temperature on the Length of the First Leaf

**Figure 1:**
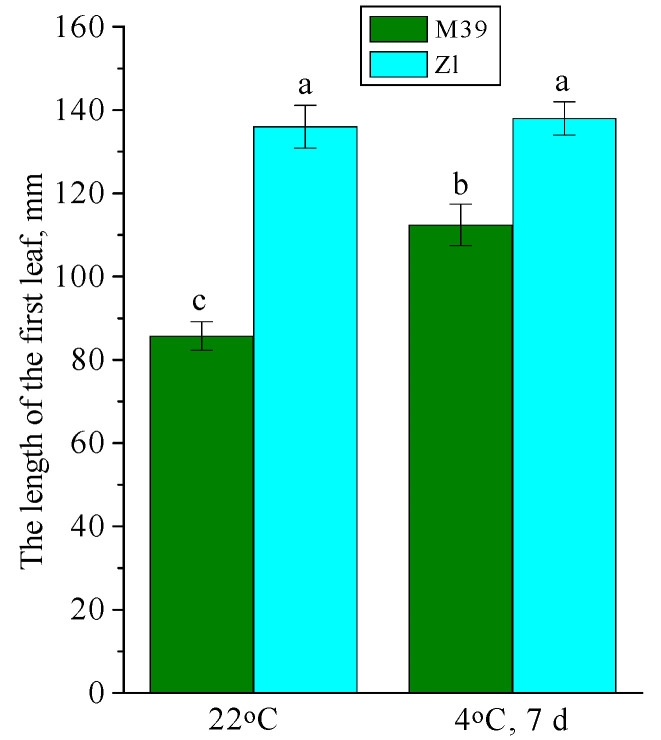
The length of the first leaf of freezing-tolerant (M39) and cold-sustainable (Zl) wheat genotypes in control conditions (22 °C) and after chilling at 4 °C during 7 days. Results presented as means of three replicates marked with the same letter were not significantly different at *p* ≤ 0.05 (Tukey’s test).

The length of the first leaf was determined in wheat seedlings at the age of 10 days, and after their 7-day cold exposure at 4 °C. It was found that wheat genotypes differed in growth intensity already at optimal temperature conditions (Figure 1). The average first leaf length of Zl seedlings was 1.5 times longer than that of M39. The growth of Zl seedlings stalled in chilling, while M39 seedlings continued to grow. In chilling the length of the first leaf in M39 seedlings was increased 1.3-fold.

### 3.2. Effect of Low Temperature on Dry Weight and Water Content

**Figure 2:**
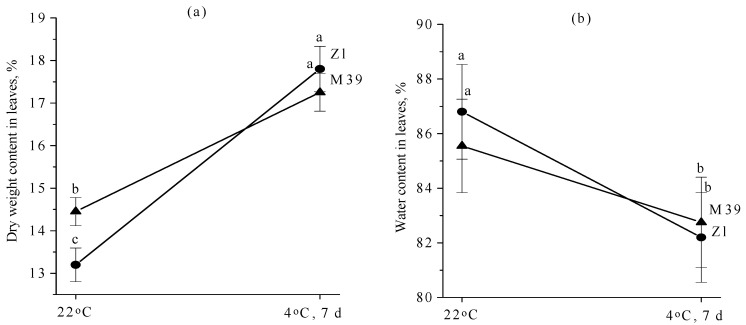
Dry weight (**a**) and water content (**b**) in leaves of freezing-tolerant (M39) and cold-sustainable (Zl) wheat genotypes in control conditions (22 °C) and after chilling at 4 °C during 7 days. Results presented as means of three replicates marked with the same letter were not significantly different at *p* ≤ 0.05 (Tukey’s test).

Under optimal conditions the dry weight of leaves of the M39 genotype was higher than that of Zl (Figure 2a). In chilling, an increase in dry weight was observed in both genotypes, but more in Zl (by 4.6%). Under these conditions, the water content of the leaves of both wheat genotypes was decreased—by 4.6% in Zl, by 2.8% in M39 (Figure 2b).

### 3.3. Effect of Low Temperature on Content of Photosynthetic Pigments

**Figure 3:**
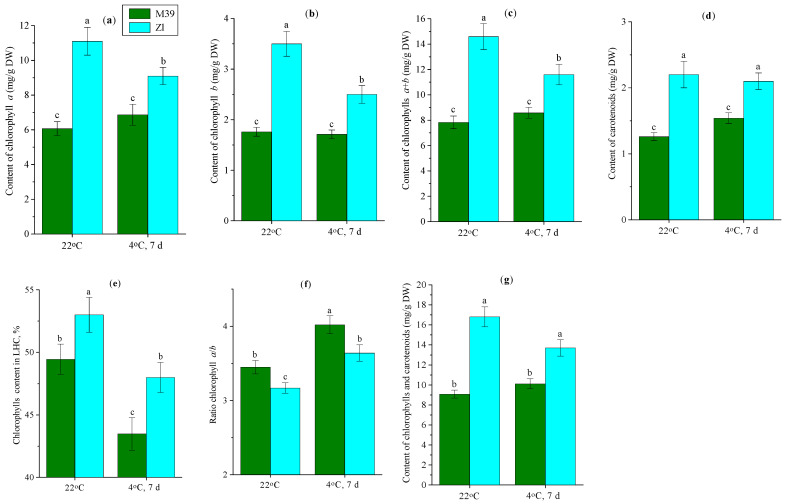
Content of chlorophyll *a* (**a**), *b* (**b**), *a* + *b* (**c**), carotenoids (**d**), chlorophyll in LHC (light-harvesting complex) (**e**), ratio *a*/*b* (**f**) and total content of pigments (**g**) in leaves of freezing-tolerant (M39) and cold-sustainable (Zl) wheat genotypes in control conditions (22 °C) and after chilling at 4 °C during 7 days. DW—dry weight. Results presented as means of three replicates marked with the same letter were not significantly different at *p* ≤ 0.05 (Tukey’s test).

One of the sensitive indicators of plant stress tolerance is the content of photosynthetic pigments in leaves. In this connection, we investigated the composition of pigments at optimum temperature and after cold exposure. It was found that wheat genotypes differed in the content of photosynthetic pigments under normal conditions (Figure 3). Leaves of M39 plants contained fewer chlorophylls *a* and *b* and carotenoids compared to the Zl genotype. In addition, the sum of chlorophylls *a* + *b* and total content of pigments in leaves of Zl were 86% and 70% higher than those of M39, respectively. Under optimal conditions, the chlorophyll content in the light-harvesting complex (LHC) in leaves of Zl plants was 3.6% higher than that of M39. At the same time, the chlorophyll ratio *a*/*b* in M39 was higher than in Zl.

A decrease in the content of photosynthetic pigments was observed in the Zl genotype under chilling, whereas an increase was observed in M39 (Figure 3). In chilling, in genotype Zl the content of chlorophyll *a* was decreased by almost 20% and chlorophyll *b* by almost 30%. Cold exposure of the plants increased the carotenoid content in the M39 genotype by more than 20%, but did not affect the carotenoid content in the Zl genotype. In both genotypes after low-temperature exposure the chlorophyll content in the LHC was decreased and the chlorophyll *a*/*b* ratio was increased.

### 3.4. Effect of Low Temperature on Intensity of LPO

**Figure 4:**
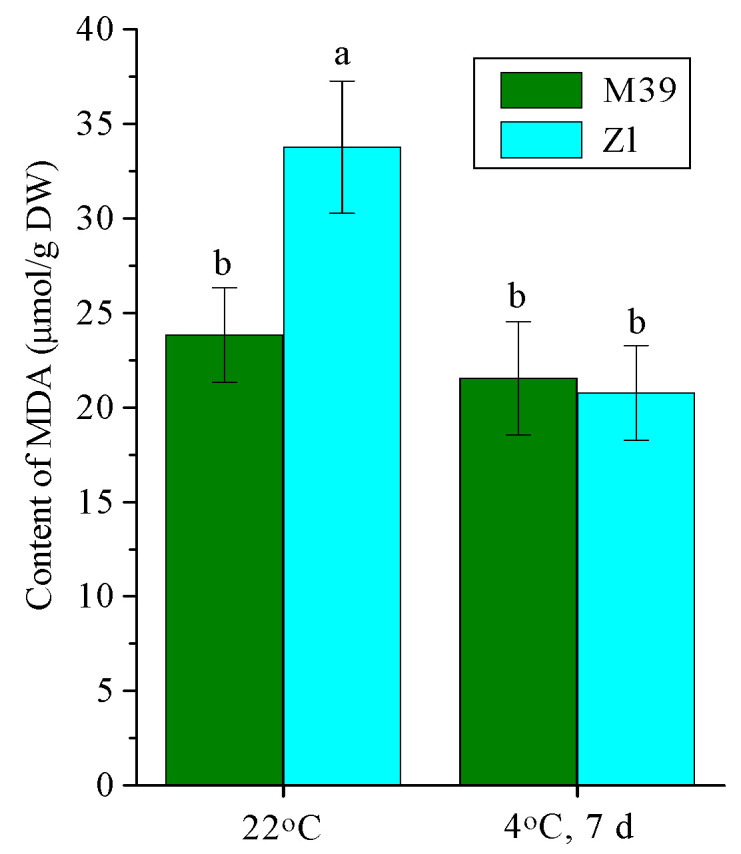
Content of MDA (malondialdehyde) in leaves of freezing-tolerant (M39) and cold-sustainable (Zl) wheat genotypes in control conditions (22 °C) and after chilling at 4 °C during 7 days. DW—dry weight. Results presented as means of three replicates marked with the same letter were not significantly different at *p* ≤ 0.05 (Tukey’s test).

Under optimal temperature conditions the content of MDA, one of the end products of membrane LPO, was lower in leaves of the M39 genotype than in Zl (Figure 4). Chilling did not change the MDA level in M39, whereas in Zl the intensity of LPO was decreased.

### 3.5. Effect of Low Temperature on the Content of Primary Metabolites

#### 3.5.1. The Total Soluble Protein

**Figure 5:**
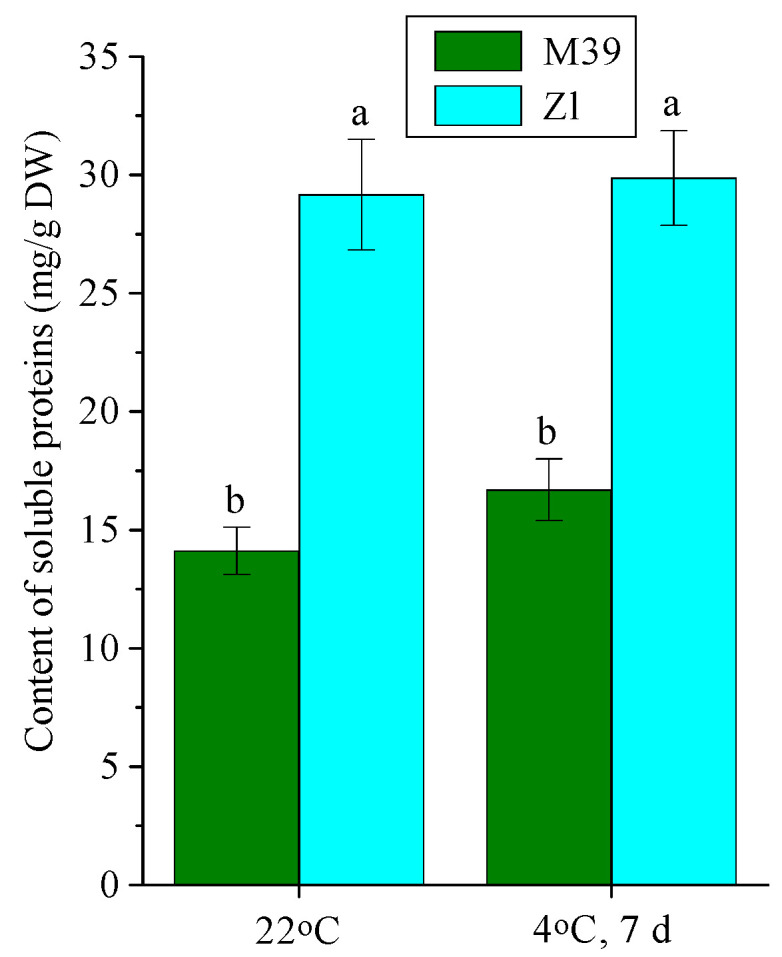
Content of soluble proteins in leaves of freezing-tolerant (M39) and cold-sustainable (Zl) wheat genotypes in control conditions (22 °C) and after chilling at 4 °C during 7 days. DW—dry weight. Results presented as means of three replicates marked with the same letter were not significantly different at *p* ≤ 0.05 (Tukey’s test).

The protein content in leaves of genotype Zl was two times higher than that of M39 under optimal temperature conditions (Figure 5). Chilling did not change the protein content in both genotypes.

#### 3.5.2. Soluble Sugars

**Figure 6:**
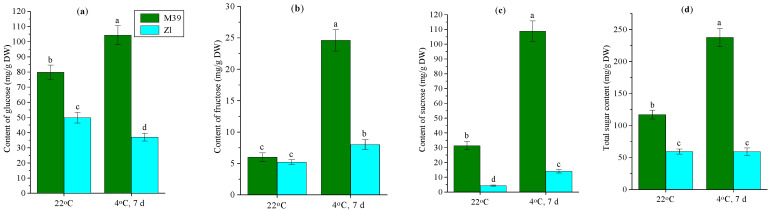
Content of soluble sugars—glucose (**a**), fructose (**b**), sucrose (**c**) and totan content of sugars (**d**) in leaves of freezing-tolerant (M39) and cold-sustainable (Zl) wheat genotypes in control conditions (22 °C) and after chilling at 4 °C during 7 days. DW—dry weight. Results presented as means of three replicates marked with the same letter were not significantly different at *p* ≤ 0.05 (Tukey’s test).

Wheat genotypes differed significantly in the content of soluble sugars (glucose, fructose, and sucrose) under optimal temperature conditions (Figure 6). Leaves of M39 seedlings contained almost two times more sugars (almost 40% more glucose and seven times more sucrose) than Zl leaves (Figure 6a,c,d). The genotypes did not differ in leaf fructose content (Figure 6b).

Our studies showed that cold exposure of plants promoted the accumulation of sugars in the leaves of genotype M39 but not Zl (Figure 6). In M39, the content of glucose was increased by 30%, fructose by four times, and sucrose by more than three times in chilling (Figure 6a–c). At the same time, in Zl against the background of a 25% decrease in glucose content, the level of fructose was increased by one and a half times, and sucrose by more than three times. However, the total content of sugars in Zl remained the same as before the exposure of the plants to cold (Figure 6d).

### 3.6. Effect of Low Temperature on the Expression of COR-Genes and Genes Encoding RuBisCo

**Figure 7:**
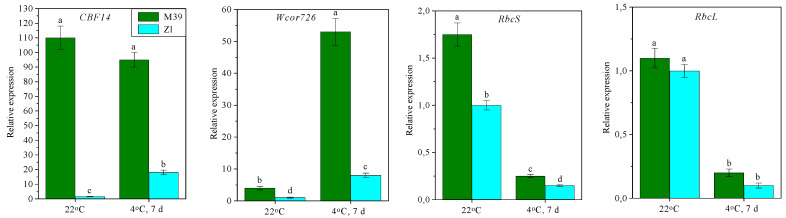
Relative expression of *COR* genes (*CBF14*, *Wcor726*) and genes, encoding small (*RbcS*) and large (*RbcL*) RuBisCo subunits in leaves of freezing-tolerant (M39) and cold-sustainable (Zl) wheat genotypes in control conditions (22 °C) and after chilling at 4 °C during 7 days. Results presented as means of three replicates marked with the same letter were not significantly different at *p* ≤ 0.05 (Tukey’s test).

Wheat genotypes differed in the expression level of genes encoding defense proteins. The expression level of *Wcor726* and *CBF14* genes was higher in the M39 genotype than in Zl (Figure 7). Low-temperature exposure of M39 seedlings increased the expression level of the Wcor726 gene but did not affect the expression level of the CBF14 gene. At the same time, in genotype Zl the expression levels of Wcor726 and CBF14 genes were sharply increased during cold exposure.

Under cold treatment of seedlings, the expression levels of RbcS and RbcL genes, encoding small and large subunits of RuBisCo, were decreased, especially in genotype Zl (9–10 times) (Figure 7).

### 3.7. Freezing Tolerance of Wheat Genotypes

The degree of freezing tolerance of wheat genotypes was evaluated by the survival of seedlings after test freezing. The percentage of undamaged seedlings was determined after 24 h of freezing at 0 °C, −3 °C, −5 °C, and −7 °C. Both control plants (grown at 22 °C) and plants after chilling (4 °C, 7 d) were frozen. The results showed that at 0 °C all wheat plants were alive (Table 2). A freezing temperature of −3 °C decreased the survival rate of seedlings, especially control Zl plants (by 85%). At the same time, there was a high percentage of survival in chilling plants (97% in the M39 genotype and 60% in the Zl genotype). Exposure of plants at −5 °C resulted in the death of all control plants in both wheat genotypes. The percentage of plants surviving chilling also decreased, but to a greater extent in genotype Zl (by 93%). A further decrease in the freezing temperature to −7 °C resulted in the death of all plants of genotype Zl, while the survival rate of M39 plants was 15%.

## 4. Discussion

In response to low temperatures, numerous physiological and biochemical changes occur in plant cells. Numerous defensive mechanisms related to the alteration of main metabolic components are triggered during freezing. The process of low-temperature-tolerance formation is actively studied in different wheat genotypes both in the field and in laboratory experiments [18,46,47]. Our study revealed the peculiarities of cold adaptation strategy of freezing-tolerant and cold-sustainable wheat genotypes. A comparative analysis of changes in growth intensity, content of photosynthetic pigments, and key primary metabolites—soluble proteins and sugars—in two wheat genotypes in optimal conditions and after chilling (4 °C, 7 d) was carried out.

This cold exposure did not induce low-temperature stress in wheat genotypes. Data for the determination of LPO intensity indicated the absence of oxidative stress—the MDA level did not increase after prolonged chilling of both genotypes (Figure 4). At the same time, cold exposure increased the tolerance of both genotypes to freezing; the M39 genotype showed a higher freezing tolerance than Zl. Hence, we used the hardening low-temperature regime.

The results of our studies showed an important role of soluble sugars (glucose, fructose, and sucrose) in increasing tolerance to low temperatures in the freezing-tolerant genotype M39. The content of sugars in the leaves of M39 was increased twofold during chilling (Figure 6). On the contrary, in cold-sustainable genotype Zl the content of soluble sugars in leaves did not accumulate during low-temperature adaptation. Soluble sugars play a significant role in stabilizing the pro-/antioxidant balance in wheat cells. Sugars are considered as components of the non-enzymatic antioxidant defense system of plants [15,48]. Soluble sugars interact with ROS both directly and indirectly, by enhancing the expression of genes encoding low-molecular-weight antioxidants [49]. Sucrose was shown to be a more active interceptor of ROS than glucose and fructose [50].

It is known that, at subzero temperatures, water freezing occurs in plant tissues, which leads to their damage and plant death [51,52]. The tolerance of cereals to low temperatures is based on the water-holding capacity of cells [53]. Zhang et al. [25] indicated that cold tolerance of wheat genotypes may be connected with effective osmoregulation ability. Our studies showed that leaf tissues of both wheat genotypes lost water during prolonged cold exposure (Figure 2). The most severe water loss was observed in cold-sustainable genotype Zl. Soluble sugars increase the water-holding capacity of cells. They are known to increase the osmotic potential of the cell, thereby reducing the freezing temperature of the solution at which ice nucleation occurs. It is hypothesized that, due to the presence of hydroxyl groups, sugar molecules bind water molecules by hydrogen bonds and some of the intracellular water is retained in a colloidal-bound form. Consequently, such water cannot participate in chemical reactions or be transported through the plant, but due to it the freezing point of the solution is reduced [54]. Soluble sugars can stabilize the structure and fluidity of cell membranes [55] in chilling due to their interaction with lipids and proteins [56]. Soluble carbohydrates are a central resource in plants [57]. They are signaling molecules, precursors of metabolic processes, serve as osmoprotectants under stress conditions, and are involved in the removal of ROS [58]. In plants, sucrose is the major compatible osmolyte that accumulates in cells. Genes associated with sucrose synthesis are modulated by exposure to environmental factors [59].

The ability of plants to survive in low-temperature conditions depends largely on their photosynthetic activity. Photosynthesis is the main target for low temperatures [60]. The chloroplasts act as sensors of environmental changes and complex networks of plastid signals coordinate cellular activities and assist the cell during plant stress responses [61]. Considering that changes in the content of photosynthetic pigments are markers of photosynthetic apparatus damage, we determined the content of chlorophylls *a* and *b* and carotenoids in leaves of different wheat genotypes under optimal conditions and after low-temperature exposure. Our studies showed that wheat genotypes differed in photosynthetic pigment content: the M39 genotype had 80% higher total pigment content than Zl (Figure 3). In chilling, the total pigment content of M39 seedlings was not changed, whereas in the cold-sustainable genotype Zl the pigment content was decreased by almost 20%, at the expense of a decrease in chlorophyll content. The expression levels of *RbcS* and *RbcL* genes, encoding the small and large subunits of RuBisCo, were decreased to a greater extent in the cold-sustainable genotype ZL under chilling conditions. However, the total photosynthetic pigment content in leaves of genotype Zl remained 35% higher after low-temperature exposure compared with M39. It can be assumed that the enzymatic activity of chlorophyll biosynthesis was decreased in Zl leaves under prolonged low-temperature exposure, in contrast to the M39 genotype. In M39 leaves the total content of photosynthetic pigments after low-temperature exposure was the same as before chilling, indicating the stability of their photosynthetic membranes and high activity of the photosynthetic apparatus.

The chlorophyll *a*/*b* ratio is one of the important indicators used to assess photosynthetic activity under abiotic stress factors [62]. As is shown in our experiments, in optimal conditions this ratio was higher in the M39 genotype (Figure 3f). After low-temperature exposure the chlorophyll *a*/*b* ratio was increased in leaves of both wheat genotypes. This was mainly true for the M39 genotype, indicating an increase in the proportion of chlorophyll *a* in the photosystem I composition.

The phase transition temperature and fluid properties of photosynthetic thylakoid membranes depend on the sugars in the surrounding solution [63]. Consequently, the increased level of sugars in leaf tissues of M39 seedlings under low-temperature exposure conditions contributed to a better protection of the photosynthetic apparatus against ROS [64]. The high cold tolerance of the photosynthetic apparatus in the M39 genotype was also evidenced by an increase in carotenoid content after cold exposure. Although carotenoids are not involved in photosynthetic reactions, they are a major component of the photosynthetic antenna, and participate in light-energy harvesting, while providing protection of the photosynthetic apparatus from ROS-induced oxidative stress and dissipating excess light energy [65].

The integral indicator that reflects the degree of plant adaptation to the environment is growth. We found out that the length of the first leaf in 10-day-old seedlings of M39, whose tissues were enriched with sugars, was 1.5 times less than in Zl (Figure 1). It is known that the process of cold adaptation is an energy-intensive process that requires energy investments from the plant organism. Therefore, growth stops almost completely in many plants under the influence of cold. Growth inhibition is accompanied by a cardinal restructuring of the metabolism associated with the inhibition of anabolic processes [66]. Our studies showed that, during prolonged exposure to low temperature, the growth of Zl seedlings was completely inhibited, whereas M39 seedlings continued to grow (Figure 1). Thus, seedlings of the M39 genotype were more adapted to low temperatures, as their growth continued even under cold conditions. Apparently, due to continued photosynthesis, sugars were accumulated and high cold tolerance was maintained. Molecules of low-molecular-weight sugars are rich in energy, and therefore they are the main substrates for cellular respiration, synthesis of stress proteins, and other primary metabolites under conditions of low-temperature exposure [67].

High cold tolerance is maintained by a large number of *COR*-genes [68,69]. Some of the main products of *COR*-genes are dehydrins, which belong to group II proteins of late embryogenesis (LEA) [70,71]. Analysis of the expression of some *COR*-genes in leaves revealed their important role in enhancing low-temperature tolerance in wheat. In both genotypes, the expression level of the *Wcor726* gene was increased during low-temperature exposure of seedlings, especially in the M39 genotype (Figure 7). *Wcor726* encodes WCOR 726 dehydrin, which refers to Wheat Cold Specific family proteins important in plant cold and freezing adaptation. In addition, regulation of *COR*-genes expression by sucrose is possible [57].

It was previously reported that the CBF (C-repeat Binding Factor) transcription factor subfamily controls the manifestation of *COR*-genes [72]. For example, among 114 metabolites that were synthesized in response to low temperature, 90% were increased by *CBF3* overexpression [73]. Studies showed that more cold-tolerant plants had higher expression levels of *CBF* genes [74], among which *CBF12*, *CBF14*, and *CBF15* played a major role [75]. We demonstrated that the expression level of the *CBF14* gene was initially much higher in freezing-tolerant genotype M39 (Figure 7). At the same time, the expression level of *CBF14* was increased more than 30-fold in genotype Zl under low-temperature conditions, but still remained 5-fold lower than in M39.

We established an important role of soluble protein accumulation in the cold adaptation of wheat, and, first of all, in cold-sustainable genotype Zl. It is important to note that already at optimum temperature Zl leaves contained two times more proteins than M39 and continued to maintain such a high level of proteins after cold exposure (Figure 5). The protein content of leaves of the freezing tolerant genotype M39 was 2-fold lower, both under optimal conditions and after low-temperature exposure. Consequently, the leaves of cold-sustainable genotype Zl are constitutively enriched in protein. Thus, the content of soluble proteins in wheat leaves is genotype-dependent. In the science literature, there are data on the increase in protein content in the leaves of 14-day-old seedlings of cold-resistant wheat genotype Shixin 828 in chilling (−8 °C, 5 h) [76]. Other authors also showed the induction of cold-regulated and antifreeze proteins during low-temperature adaptation of cold-tolerant wheat genotypes [46]. In addition, during cold adaptation of diploid wild wheat (*Triticum urartu* L.), there was also an increase in LEA and dehydrin proteins [47].

## 5. Conclusions

Experiments confirmed that the freezing-tolerant wheat genotype M39 had a significantly higher tolerance to low temperatures than the cold-sustainable genotype Zl. It is concluded that wheat genotypes contrasting in resistance to low temperatures differ in the strategy of cold adaptation, and primary metabolites—sugars and proteins—play an important role in this process. Plants of the low-temperature-sustainable genotype Zl differed from the freezing-tolerant genotype M39 by high growth rate, increased content of photosynthetic pigments and soluble proteins, and decreased content of soluble sugars in optimal conditions. In plants of freezing-tolerant genotype M39, the strategy of adaptation to prolonged low-temperature exposure was aimed at modifying carbohydrate metabolism—the accumulation of soluble sugars in tissues and, to a lesser extent, increasing the content of soluble proteins. The accumulation of sugars was not observed in wheat of cold-sustainable genotype Zl during chilling, but a high content of soluble proteins was maintained both under optimal conditions and after cold exposure. The adaptation strategy of wheat genotype M39 was related to the high expression of genes encoding COR proteins (*Wcor726*, *CBF14*). The data improve our knowledge of physiological and biochemical mechanisms of wheat cold adaptation. These data can serve as a reference for practical phytobiotechnology for screening wheat genotypes tolerant to low temperatures and for obtaining new cold- and freezing-tolerant wheat genotypes.

## Figures and Tables

**Table 1:** Primers for qRT-PCR analysis.

Gene Name	Primer	Primer Sequences
*TaRP15*	Forward	TCATTGTGGAGGACTCGTGG
	Reverse	GCAGACATAGCCCACACAT
*Wcor726*	Forward	ACTGGAATGACCGGCTCG
	Reverse	TGTCCCGACTTCCCGTAGTT
*CBF14*	Forward	ACAACCGATGACGAGAAGGAAA
	Reverse	AACCAGTGCTCATTCAACAGC
*RbcS*	Forward	GGATTCGACAACATGCGCCAGG
	Reverse	ATATGGCCTGTCGTGAGTGAGC
*RbcL*	Forward	ACCATTTATGCGCTGGAGAGACC
	Reverse	CAAGTAATGCCCCTTGATTTCACC

**Table 2:** Survival rate (%) of control and chilling (4 °C, 7 d) wheat seedlings of genotypes Moskovskaya 39 (M39) and Zlata (Zl) after freezing testing.

Freezing	M39		Zl	
	22 °C	4 °C, 7 d	22 °C	4 °C, 7 d
0 °C, 24 h	100 ^a^	100 ^a^	100 ^a^	100 ^a^
−3 °C, 24 h	35 ± 2 ^c^	97 ± 3 ^a^	15 ± 2 ^d^	60 ± 3 ^b^
−5 °C, 24 h	0	65 ± 4 ^b^	0	7 ± 2 ^e^
−7 °C, 24 h	0	15 ± 2 ^d^	0	0

Results marked with the same letter were not significantly different at *p* ≤ 0.05 (Tukey’s test).

## Data Availability

The data presented in this study are available on request from the corresponding author. The data are not publicly available due to privacy.
